# Brain Renin–Angiotensin System at the Intersect of Physical and Cognitive Frailty

**DOI:** 10.3389/fnins.2020.586314

**Published:** 2020-09-30

**Authors:** Caglar Cosarderelioglu, Lolita S. Nidadavolu, Claudene J. George, Esther S. Oh, David A. Bennett, Jeremy D. Walston, Peter M. Abadir

**Affiliations:** ^1^Division of Geriatrics, Department of Internal Medicine, Ankara University School of Medicine, Ankara, Turkey; ^2^Division of Geriatric Medicine and Gerontology, Johns Hopkins University School of Medicine, Baltimore, MD, United States; ^3^Division of Geriatrics, Department of Medicine, Albert Einstein College of Medicine, Montefiore Medical Center, Bronx, NY, United States; ^4^Rush Alzheimer’s Disease Center, Rush University Medical Center, Chicago, IL, United States

**Keywords:** renin–angiotensin system, RAS, brain, neurodegenerative diseases, neuroinflammation, oxidative stress, physical and cognitive frailty, aging

## Abstract

The renin–angiotensin system (RAS) was initially considered to be part of the endocrine system regulating water and electrolyte balance, systemic vascular resistance, blood pressure, and cardiovascular homeostasis. It was later discovered that intracrine and local forms of RAS exist in the brain apart from the endocrine RAS. This brain-specific RAS plays essential roles in brain homeostasis by acting mainly through four angiotensin receptor subtypes; AT_1_R, AT_2_R, MasR, and AT_4_R. These receptors have opposing effects; AT_1_R promotes vasoconstriction, proliferation, inflammation, and oxidative stress while AT_2_R and MasR counteract the effects of AT_1_R. AT_4_R is critical for dopamine and acetylcholine release and mediates learning and memory consolidation. Consequently, aging-associated dysregulation of the angiotensin receptor subtypes may lead to adverse clinical outcomes such as Alzheimer’s disease and frailty via excessive oxidative stress, neuroinflammation, endothelial dysfunction, microglial polarization, and alterations in neurotransmitter secretion. In this article, we review the brain RAS from this standpoint. After discussing the functions of individual brain RAS components and their intracellular and intracranial locations, we focus on the relationships among brain RAS, aging, frailty, and specific neurodegenerative diseases, such as Alzheimer’s disease, Parkinson’s disease, and vascular cognitive impairment, through oxidative stress, neuroinflammation, and vascular dysfunction. Finally, we discuss the effects of RAS-modulating drugs on the brain RAS and their use in novel treatment approaches.

One of the biggest challenges of healthcare in the 21st century is the care of older adults suffering from cognitive impairment and frailty because of their high medical, economic, psychological, and social burden. Frailty, among the most common geriatric syndromes, is characterized by failure of homeostatic mechanisms, diminished physical function, and reduced age-related physiologic reserve leading to decreased ability to cope with stressors and increased vulnerability to adverse outcomes (Fried et al., 2001; Ma and Chan, 2020). There is growing evidence linking frailty and cognitive impairment (Boyle et al., 2010; Subra et al., 2012; Robertson et al., 2013; Ma et al., 2019; Wallace et al., 2019). Although studies in epidemiology and pathology have shown strong associations between frailty and cognitive impairment, the association’s biological basis still remains elusive. One study found shared pathologies associated with both (Buchman et al., 2014). There are likely additional shared mechanisms linking the two, including inflammation, oxidative stress, mitochondrial damage, and cellular regeneration failure. The identification of this biological link can lead to new preventive and therapeutic interventions for both conditions. In this regard, the following section introduces the renin–angiotensin system, a critical hormonal system that can provide further insight into the biological links between frailty and cognition.

## Introduction: An Overview of the Classical and Local Renin–Angiotensin System

The renin–angiotensin system (RAS) was first identified as part of the endocrine system, which regulates water and electrolyte balance, systemic vascular resistance, aldosterone release, and cardiovascular homeostasis. After the discovery of renin (Tigerstedt and Bergman, 1898), it took 40 years to isolate the other component of the RAS, angiotensin, by two groups simultaneously (Braun-Menendez et al., 1940; Page and Helmer, 1940). The classical RAS is activated by the release of renin from juxtaglomerular cells of renal afferent arterioles into the circulation. As the first and rate-limiting step, renin converts the precursor molecule angiotensinogen to angiotensin I (Ang I), which is then transformed to angiotensin II (Ang II) by angiotensin-converting enzyme (ACE), localized mostly in the endothelial cells of the lungs. Within this system, Ang II is a primary bioactive product, leading to antagonistic effects including vasoconstriction and vasodilation by, respectively, binding to angiotensin II type 1 receptor (AT_1_R) and angiotensin II type 2 receptor (AT_2_R) (Griendling et al., 1993; Unger et al., 1996; Vajapey et al., 2014).

**FIGURE 1:**
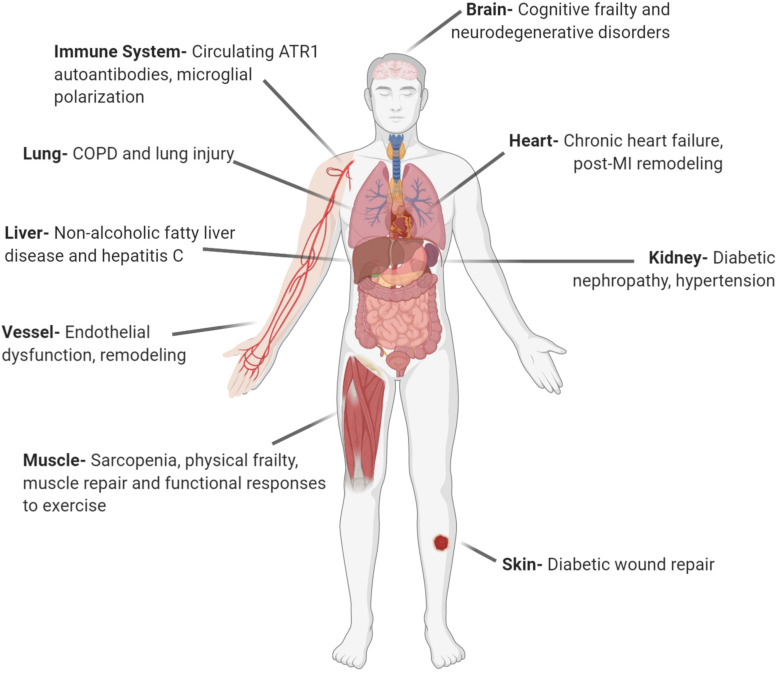
Local angiotensin systems have broad clinical relevance. AT_1_R, angiotensin receptor type 1; COPD, chronic obstructive pulmonary disease; MI, myocardial infarction (Created with BioRender.com).

Beyond the classical endocrine (circulating) RAS, additional research identified the autocrine (cell to the same cell type) and paracrine (cell to different cell type) effects of RAS. Further, it has been shown that RAS can be locally synthesized and act in many tissues including endothelial cells, adrenal and pituitary glands, testis, ovary, kidney, heart, and eye (Dzau, 1988; Paul et al., 2006; Vajapey et al., 2014). This form of RAS was termed local or tissue RAS. The broad clinical relevance of local RAS is shown in Figure 1. Among the local RASs, brain RAS (b-RAS), discovered by Ganten et al. (1971) has a particular importance, as systemic RAS components cannot access most brain regions because of the blood–brain barrier (BBB) (Wright and Harding, 2013). Besides these tissue-level RAS, subcellular functional units of RAS in organelles such as mitochondria and nuclei were revealed by different research groups (Abadir et al., 2011, 2012; Gwathmey et al., 2012).

Further research on b-RAS has shown that it has complicated effects on the central nervous system beyond its well-known roles, including sodium retention, vascular, and blood pressure control. There is growing evidence of b-RAS’ impact on oxidative stress, endothelial dysfunction, microglial polarization, neuroinflammation, brain homeostasis, alterations in neurotransmitter secretion, cognition, and even aging and frailty (Rodriguez-Pallares et al., 2008; Abadir, 2011; De Silva and Faraci, 2013; Wright and Harding, 2013; Labandeira-Garcia et al., 2017; Forrester et al., 2018). The use of classical RAS-acting drugs like angiotensin receptor blockers (ARB) or ACE inhibitors (ACEI) for the regulation of b-RAS has long been under investigation.

After reviewing the structure and function of b-RAS, we consider its roles in aging and neurodegenerative diseases with respect to oxidative stress, neuroinflammation, and vascular dysfunction. Finally, we summarize the effects of angiotensin system modulators on b-RAS and their therapeutic potential.

## Structure of the Brain Renin–Angiotensin System

There are two main RASs in the brain: circulating and local (Saavedra, 2005; Grobe et al., 2008). Circulating RAS exerts its effect via the circumventricular organs, which are regions of the brain that lack BBB and project to nuclei in the hypothalamus and medulla (Lenkei et al., 1997; Gao and Zucker, 2011). In contrast, b-RAS, which is the independent local RAS of the brain, can synthesize all components of the circulatory RAS (Harding et al., 1988; Wright and Harding, 2013). Despite a few recent studies with conflicting results (van Thiel et al., 2017), it is generally accepted that there is *de novo* production of RAS components as well as active RAS genes and their promoter regions in the brain (Fuxe et al., 1980; Hermann et al., 1987; Harding et al., 1988; Keisuke et al., 2017). In a double-transgenic mouse, it was demonstrated that there are specific renin-expressing cells near angiotensinogen-expressing cells, specifically within the rostral ventrolateral medulla (RVLM) (Lavoie et al., 2004a,b).

### Angiotensin Ligands and Peptidases Within the Brain

**FIGURE 2:**
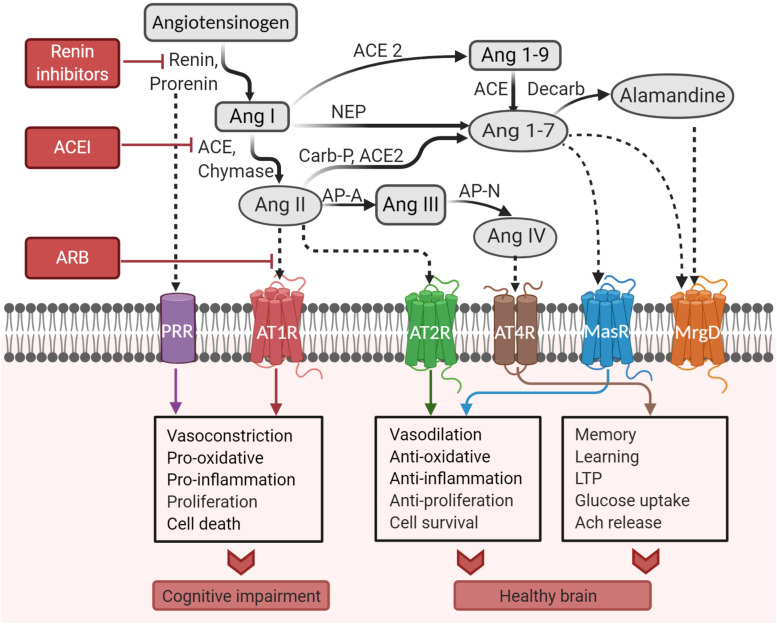
Pathways of the brain renin–angiotensin system. ACE, angiotensin-converting enzyme; ACEI, angiotensin-converting enzyme inhibitor; Ang, angiotensin; AP-A, aminopeptidase A; AP-N, aminopeptidase N; ARB, angiotensin receptor blocker; AT_1_R, angiotensin II type I receptor; AT_2_R, angiotensin II type 2 receptor; AT_4_R, angiotensin IV receptor; Carb-P, carboxypeptidase-P; decarb, decarboxylase; LTP, long-term potentiation; MasR, Mas receptor; MrgD, Mas-related-G protein-coupled receptor; NEP, neutral endopeptidase; PRR, prorenin receptor (Created with BioRender.com).

Angiotensinogen is mainly produced and secreted from astrocytes within the brain to be cleaved into various neuroactive angiotensin peptides (Stornetta et al., 1988; Milsted et al., 1990). Conversion of angiotensinogen to the decapeptide Ang I is catalyzed by renin. Then, the zinc metalloprotease ACE hydrolyzes the carboxy-terminal dipeptide of Ang I to form the octapeptide Ang II (Wright and Harding, 2013). Cathepsin and chymase can also hydrolyze Ang I (Unger and Li, 2004; Mogi et al., 2012). Glutamyl aminopeptidase A (AP-A) cleaves the aspartate residue at the N-terminal of Ang II to form the heptapeptide angiotensin III (Ang III), which is then converted to the hexapeptide angiotensin IV (Ang IV) by alanyl aminopeptidase N (AP-N) cleaving arginine at the N-terminal. Ang IV can be further converted to Ang (3–7) by carboxypeptidase P and prolyl oligopeptidase. Alternatively, Ang II can be converted to Ang (1–7) by carboxypeptidase P and ACE2, which is an isoform of ACE (Wright and Harding, 2013). ACE2 can also convert Ang I to Ang (1–9). Ang (1–7) can be converted from Ang (1–9) by ACE or from Ang I by neutral endopeptidase (Jiang et al., 2013). A recently discovered component of RAS is alamandine, which is formed by the decarboxylation of Ang (1–7) (Lautner et al., 2013). Alternatively, alamandine can be generated by ACE2 cleaving angiotensin A, which is obtained by decarboxylation of Ang II (Lautner et al., 2013). Ang II, Ang (1–7), and Ang IV are the main neuroactive angiotensin peptides that trigger signal transduction as they bind to their cognate receptors. The entire pathway is illustrated in Figure 2.

In classical RAS, renin is formed by the cleavage of prorenin. However, renin activation that generally occurs in secretory granules of renal juxtaglomerular cells is not as common in extrarenal tissues such as the brain (Hirose et al., 1981). Renin concentration is low within neurons and astrocytes (Grobe et al., 2008; Bodiga and Bodiga, 2013). However, prorenin receptor (PRR), which activates prorenin without prosegment removal, is highly expressed in neurons and some microglial cells in several cardiovascular brain regions such as the subfornical organ (SFO), paraventricular nucleus (PVN), the nucleus of the solitary tract (NTS), and RVLM as well as non-cardiovascular brain regions such as brain cortex and basal ganglia (Nguyen et al., 2002; Valenzuela et al., 2010; Li et al., 2012a,b; Garrido-Gil et al., 2013b, 2017; Xu et al., 2016). Overactivation of this system can lead to cognitive impairment by the activation of the Ang II/AT_1_R axis (Wright and Harding, 2011; Bodiga and Bodiga, 2013). Besides increasing the catalytic activity of prorenin and renin to hydrolyze angiotensinogen into angiotensin, PRR has Ang II-independent effects (Nguyen and Contrepas, 2008; Shan et al., 2008). In this context, PRR can initiate its own signaling pathway and induce its pro-oxidative effects (Labandeira-Garcia et al., 2017). Although it was shown that there is a relationship between PRR and neural development (Trepiccione et al., 2016), RAS-independent functions of PRR within the brain are not yet clear (Nakagawa et al., 2020).

Furthermore, another brain-specific renin isoform called renin-b was also discovered (Lee-Kirsch et al., 1999; Sinn and Sigmund, 2000). While renin-b was initially believed to be effective in the intracellular generation of Ang II, it is now thought to be more involved in intracellular regulation of b-RAS (Grobe et al., 2008; Keisuke et al., 2017).

### Angiotensin Receptors and Their Locations Within the Brain

Ang II acts through two receptor subtypes: AT_1_R and AT_2_R, both of which are members of the G-protein-coupled receptor (GPCR) family (Miura et al., 2013). Ang III also binds to these receptors although forms higher-affinity bonds with AT_2_R and lower-affinity bonds with AT_1_R compared to Ang II (Wright and Harding, 2011). Human AT_1_R contains 359 amino acids and has a molecular mass of 41 kDa, while AT_2_R, which is 34% identical to AT_1_R, consists of 363 amino acids and also has a mass of 41 kDa (de Gasparo et al., 2000; Zhang et al., 2017). Binding of Ang II to AT_1_R, accompanied by changes in its transmembrane 3–6 conformation, induces cell signaling (Inoue et al., 1997; Ghanouni et al., 2001; Miura et al., 2013). AT_1_Rs and AT_2_Rs were identified mostly in the cortex, hippocampus, and basal ganglia in humans and many animals (Lenkei et al., 1996; Rodriguez-Pallares et al., 2008; Joglar et al., 2009; Valenzuela et al., 2010; Garrido-Gil et al., 2013b, 2017; Jackson et al., 2018). There are two types of AT_1_Rs: AT1A and AT1B. The AT1A receptor is mainly expressed in brain regions contributing to homeostatic regulation of blood pressure and electrolyte balance (Jöhren et al., 1995; MacGregor et al., 1995; Lenkei et al., 1997), whereas the AT1B receptor is seen in structures involved in memory and higher brain functions such as the cerebral cortex and hippocampus (Jöhren et al., 1995). AT_2_R is present in brain regions involved in learning and memory, particularly the hippocampus, cingulate cortex, lateral septum, and locus coeruleus, but also the superior colliculus, thalamic and subthalamic nuclei, and inferior olive (Millan et al., 1991; Lenkei et al., 1996). AT_1_Rs and AT_2_Rs have been identified mostly in neurons, astrocytes, oligodendrocytes, and microglia of those regions (Tsutsumi and Saavedra, 1991; Fogarty and Matute, 2001; Rodriguez-Pallares et al., 2008; Joglar et al., 2009; Garrido-Gil et al., 2013b, 2017). However, a few studies could not identify AT_1_Rs in microglia (Lenkei et al., 1997; Benicky et al., 2009) likely because of their methodology and the fact that AT_1_Rs are undetectable in microglia’s non-activated state and then upregulated only upon activation (Lanz et al., 2010; Rodriguez-Perez et al., 2015; Sun et al., 2015; Labandeira-Garcia et al., 2017). A more recent investigation of cellular locations of AT_1_R and AT_2_R under normal and hypertensive conditions showed that AT_1_Rs and AT_2_Rs are localized to neurons rather than astrocytes and microglia in PVN, NTS, arcuate nucleus, dorsomedial hypothalamus, area postrema, median preoptic nucleus, SFO, and organum vasculosum of the lamina terminalis (Sumners et al., 2020).

Another receptor is angiotensin receptor 4 (AT_4_R), to which Ang IV binds. However, in high concentrations of Ang IV, it can bind to AT_1_R (Jackson et al., 2018). Unlike other angiotensin receptors, AT_4_R is identical to insulin-regulated aminopeptidase (IRAP), which is a type 2 transmembrane protein of the gluzincin aminopeptidase family, including homologous aminopeptidases AP-A and AP-N (Lee et al., 2003; Farag et al., 2017; Wright and Harding, 2019). AT_4_Rs are more abundant in sensory and cognitive regions of the brain in contrast to AT_1_Rs that are abundant in cardiovascular and osmoregulatory areas (Wyse et al., 1995). The fact that AT_4_R and cholinergic receptors are closely localized suggests that AT_4_Rs have a potential role in learning and memory (Wilson et al., 2009). AT_4_Rs are present in many components of the central nervous system, particularly the caudate, putamen, cerebellum, anterior pituitary globus pallidus, neocortex, CA1–CA3 pyramidal layers of hippocampus, nucleus basalis of Meynert, lateral geniculate body, ventral lateral thalamic nucleus, motor neurons of the brain stem, and ventral horn of the spinal cord (Wright and Harding, 2011). At the cellular level, it is well known that AT_4_R is located on neurons (Chai et al., 2000; Albiston et al., 2001). However, there are conflicting results about their presence on astrocytes (Greenland et al., 1996; Jackson et al., 2018; O’Connor and Clark, 2018). In a recent study, the presence of IRAP/AT_4_R was shown in both pinealocytes and astrocytes in the rat pineal gland (Abrahão et al., 2019).

Ang (1–7) binds to Mas receptors (MasRs) with the highest affinity, but it can also bind to Mas-related-G protein-coupled receptors (MrgDs) and AT_2_Rs with low affinity (Elased et al., 2008; Bernstein et al., 2014; Tetzner et al., 2016). MasRs are GPCRs with high concentrations in the brain structures associated with memory and cognition such as the hippocampus, but also the piriform cortex involved in smell (Freund et al., 2012). Although they were seen in both neuronal and glial cells, the MasR immunoreactivity was higher in neurons than in glia (Costa-Besada et al., 2018). Recently discovered MrgDs are also GPCRs, and their primary ligand is alamandine (Lautner et al., 2013). The discovery of MrgDs has added another level of complexity into RAS, and further studies are needed for a complete understanding of their roles within the brain.

Hetero-dimerizations have been observed between GPCRs of the RAS. While AT_1_R and bradykinin B2 receptor hetero-dimerization enhances G-protein activation (Quitterer et al., 2011; Miura et al., 2013), AT_2_R/AT_1_R and MasR/AT_1_R heterodimers directly antagonize and inactivate AT_1_Rs, leading to a decrease in AT_1_R signaling cascade (AbdAlla et al., 2001). Also, AT_2_Rs and MasRs may form heterodimers as they have similar signaling mechanisms, and are functionally dependent on each other (Leonhardt et al., 2017). In this regard, it was shown that MasR protein expression and mRNA levels in the substantia nigra (SN) of AT_2_R knockout (KO) mice decreased, rendering Ang (1–7) ineffective in astrocytes, whereas MasR expression did not change in AT_1_R KO mice (Villela et al., 2015; Leonhardt et al., 2017). However, notably, AT_2_R expression and ACE2 and Ang 1–7 levels decreased in the absence of the Ang II/AT_1_R axis (Villar-Cheda et al., 2010).

Angiotensin receptors can also be located intracellularly. Specifically, they have been found in the nucleus, mitochondria, neurosecretory vesicles, and the plasma membrane (Sadoshima et al., 1993; Vila-Porcile and Corvol, 1998; Sherrod et al., 2005; Peters, 2008; Abadir et al., 2011). Intracellular RAS can be regulated independently from the systemic circulation (Abadir et al., 2012). Whether circulating (classical), local, or subcellular (intracrine) RAS, the main system processes are the same (Kumar R. et al., 2012). Ang II and enzymes such as renin and ACE can be localized to cytoplasm and nuclei; ACE also has been found on the endoplasmic reticulum (Shen et al., 2008; Abadir et al., 2012).

## Functions of Renin–Angiotensin System Receptors

Here, we review the general functions of RAS receptors and discuss their cell type-specific functions within the brain.

### Prelude to the Renin–Angiotensin System Signaling and Functions

AT_1_R and AT_2_R stimulation generally lead to opposing actions. While AT_1_R mediates vasoconstriction, cellular proliferation, cell growth, and production of superoxide, AT_2_R mediates vasodilatation and has both anti-oxidant and anti-inflammatory properties (Abadir, 2011; Abadir et al., 2011).

Although Ang II can bind to both AT_1_R and AT_2_R, ACE upregulation prompts AT_1_R signaling (Jackson et al., 2018). After G-protein coupling stimulation of AT_1_R with Ang II, second messenger signaling, which consists of inositol trisphosphate, diacylglycerol, and arachidonic acid, initiates activation of downstream effectors, such as phospholipases C, A, and D. Response of these signals can differ across tissues. Protein kinase C, Akt, intracellular protein kinases (such as receptor and non-receptor tyrosine kinases), and serine/threonine kinases [such as mitogen-activated protein kinase (MAPK) family kinases] are activated by the AT_1_R signaling cascade (Forrester et al., 2018). Overactivation of AT_1_R cascade may result in hypertrophy, vascular remodeling, and hyperplasia (Suzuki et al., 2005).

The Ang II/AT_2_Rs axis signals through three major transduction pathways that seem to oppose the actions of AT_1_R. AT_2_R activates several protein phosphatases and nitric oxide (NO)/cyclic GMP system and stimulates phospholipase A2, with subsequent release of arachidonic acid (Abadir, 2011). AT_2_R also inhibits cell growth and proliferation by inhibiting autophosphorylation of insulin and epidermal growth factor receptors (Steckelings et al., 2010). Furthermore, AT_1_R blockade increases angiotensinogen and AT_2_R stimulation by inhibition of negative feedback (Carey et al., 2001). The AT_1_R blockade also increases renal NO, which is blunted by concomitant AT_2_R blockade (Siragy et al., 2000), suggesting AT_2_R’s role in increasing NO production via direct stimulation of NO synthase (NOS) or indirectly through bradykinin-dependent mechanisms (Abadir et al., 2003).

### Receptor Functions According to Cell Type

#### Neurons

Neurons have all types of angiotensin receptors on their cell membrane; also, they have an intracellular angiotensin system that works separately. It has been shown that neurons have intracrine RAS, including AT_1_Rs, AT_2_Rs, and MasRs in their mitochondria and nuclei (Valenzuela et al., 2016; Villar-Cheda et al., 2017; Costa-Besada et al., 2018). Although astrocytes synthesize most of the angiotensinogen within the brain, neurons can also synthesize angiotensinogen. Angiotensinogen immunoreactivity is found in the entire brain, predominantly in areas responsible for water and electrolyte balance such as the SFO, PVN, NTS, and RVLM (Grobe et al., 2008). This wide distribution of angiotensinogen in the brain shows that b-RAS is not limited to cardiovascular regulatory functions (Bodiga and Bodiga, 2013). As neurons contain RAS enzymes (e.g., intracellular renin, prorenin, etc.), angiotensinogen can be converted to active angiotensin peptides intracellularly (Lee-Kirsch et al., 1999; Abadir et al., 2012; Jackson et al., 2018).

The Ang II/AT_1_R axis produces reactive oxygen species (ROS) and causes oxidative stress by NADPH oxidase in neurons, as in other tissues. Conversely, the Ang II/AT_2_R axis and Ang (1–7)/MasR axis produces NO and inhibits superoxide increment (Costa-Besada et al., 2018). In neurons, while the Ang II/AT_1_R axis and the (Pro)renin/PRR axis have pro-oxidative and pro-inflammatory properties, the Ang II/AT_2_R axis and the Ang (1–7)/MasR axis act as the protective arm of the RAS (Costa-Besada et al., 2018). Mitochondrial AT_2_Rs are more common than AT_1_Rs in dopaminergic neurons (Valenzuela et al., 2016). Notably, even though MasR is less concentrated in mitochondria than the rest of the cell, both ACE2 and Ang (1–7) are more abundant in mitochondria (Costa-Besada et al., 2018). This study supports a recent finding suggesting that MrgDRs and AT_2_Rs are additional Ang (1–7) receptors (Tetzner et al., 2016).

In neurons, Ang II, Ang III, and AT_2_Rs are colocalized in the mitochondrial inner membrane (Abadir et al., 2011). This colocalization is important since mitochondrial NOS, a possible mitochondrial respiration regulatory enzyme, is also known to exist in the mitochondrial inner membrane (Ghafourifar and Richter, 1997). Also, it was shown that the AT_2_R agonist CGP421140 activates NO production in a dose-dependent manner, and that AT_2_R blocker PD-123319 mitigates it (Abadir et al., 2011).

As with mitochondrial RAS, nuclear RAS is essential for the regulation of oxidative stress. It maintains the balance between detrimental and protective pathways of RAS through transcription and trafficking of additional receptor types (Zawada et al., 2015; Villar-Cheda et al., 2017; Costa-Besada et al., 2018). When nuclear AT_1_Rs are activated, both oxidative and anti-oxidative mechanisms are initiated. An increase in mRNA levels of PRR, angiotensinogen, and renin are seen, leading to intracellular synthesis of more Ang II and Ang (1–7). Concomitantly, protective AT_2_R levels increase. AT_2_Rs are then delivered to different organelles such as mitochondria and cell membranes. While Ang (1–7) shows its protective effects, increased Ang (1–7) levels suppresses AT_2_R expression (Villar-Cheda et al., 2017; Costa-Besada et al., 2018). These compensatory mechanisms can be dysfunctional in aging and cognitive disorders (Labandeira-Garcia et al., 2017).

Overactivation of the Ang II/AT_1_R axis has many detrimental effects on the brain, such as hypertension, neuroinflammation, increased oxidative stress, BBB disruption, and neurotoxicity. Neuronal AT_1_R activation *in situ* or brain slices may increase the firing rate of neurons in specific brain regions, such as the SFO, PVN, and RVLM (Knowles and Phillips, 1980; Sumners et al., 2002). This effect can be reversed by losartan, an ARB (Sumners et al., 2002). The Ang II/AT_1_R axis enhances sympathetic neurotransmitter release in the central nervous system. In particular, the release of vasopressin, dopamine, and norepinephrine is facilitated (Stadler et al., 1992; Medelsohn et al., 1993; Tsuda, 2012). Also, studies have investigated the effects of the Ang II/AT_1_R axis b-RAS on inhibitory GABA and excitatory glutamate transmitters. It was shown that Ang II/AT_1_R axis decreases GABA and enhances glutamate release (Tedesco and Ally, 2009; Fujita et al., 2012; Tsuda, 2012). Notably, there is evidence that activation of AT_2_R and MasR functions in opposition to AT_1_R’s neurosecretory effect (Tsuda, 2012; de Kloet et al., 2016).

In contrast to the effects of Ang II/AT_1_R signaling, Ang II induces neuroprotective mechanisms, NO production, neurite outgrowth, and brain development through AT_2_R activation, thus improving cognitive function (Farag et al., 2017). In this regard, impaired AT_2_R signaling results in AT_1_R-mediated oxidative stress and neuroinflammation, which may lead to impaired cognition. For example, within the hippocampus, decreased AT_2_R activation was shown to cause dendritic spine abnormalities and spatial memory deficits (Maul et al., 2008). As another neuroprotective mechanism, AT_2_R activation improved neuronal survival and neurological deficits via increased VEGF production after ischemic injury in rodents (Mateos et al., 2016). AT_2_R activation also enhances the repair of damaged DNA and differentiation in the nervous system through induction of methyl methanesulfonate sensitive 2, a ubiquitin-conjugating enzyme variant (Mogi and Horiuchi, 2013).

Another neuroprotective axis is Ang (1–7)/MasR, which limits the pressor, angiogenic, and proliferative actions of Ang II (Ho and Nation, 2018). This axis has both anti-oxidant and anti-inflammatory properties (Jiang et al., 2013). It has essential roles in learning, memory, neuroprotection, and cell survival (Wright et al., 2013; Farag et al., 2017). Besides, the Ang (1–7)/MasR axis is well known for promoting the production of arachidonic acid and activation of endothelial and neuronal NO synthase, which are crucial for object recognition memory and long-term potentiation (LTP) in the hippocampus and amygdala (Hellner et al., 2005; Yang et al., 2010). Indeed, deficient object recognition memory was observed in MasR KO mice (Lazaroni et al., 2012). This axis also plays a neuroprotective role against ischemic stroke by significantly increasing the density of brain capillaries, improving regional cerebral blood flow (CBF), and decreasing infarct volume and neurological deficits (Jiang et al., 2013). This role was further confirmed by a study in which the brain angiogenic effects of Ang (1–7) were attenuated with MasR antagonist A-779 (Jiang et al., 2014). A recently discovered effect of Ang (1–7)/MasR axis is an increase of glucose utilization and decrease of insulin resistance (IR) (Williams et al., 2016; Loloi et al., 2018; Wright and Harding, 2019). Consistent with this effect, chronic administration of Ang-(1–7) improved glucose tolerance in fructose-fed rats (Guimaraes et al., 2014).

As discussed above, AT_4_Rs are mostly located on neurons, specifically in the sensory and cognitive regions. One of the first studies presenting behavioral evidence for the cognitive effects of AT_4_Rs showed improvement in the learning and memory functions of rats with scopolamine-induced deficits in the circular water maze task by using an Ang IV analog intracerebroventricularly (Pederson et al., 1998). Previous work suggested that actions of Ang IV are partially mediated by IRAP which codistributes with the insulin-regulated glucose transporter (GLUT 4) (Albiston et al., 2001, 2003). In this context, AT_4_Rs show some of their effects by inhibiting the catalytic activity of IRAP (Lew et al., 2003; Singh and Karnik, 2016; Abrahão et al., 2019). With this inhibition, Ang IV extends the half-life of several pro-cognitive endogenous peptides such as vasopressin, oxytocin, somatostatin, and endothelial NOS, which have been shown to enhance memory consolidation and retrieval. A parallel AT_4_R/IRAP mediated pathway that enhances memory is through neuronal glucose uptake via regulation of GLUT4 vesicular trafficking (Chai et al., 2004; Fernando et al., 2008; Wright and Harding, 2011; Farag et al., 2017). Moreover, AT_4_R activation induces a non-*N*-methyl-D-aspartate (non-NMDA)-dependent form of LTP via increases in intracellular calcium influx (Davis et al., 2006). Regarding the cognitive effects of Ang IV, IRAP inhibitors are shown to improve memory (De Bundel et al., 2009; Albiston et al., 2011; Mountford et al., 2014). Another suggested neuroprotective pathway of Ang IV/AT_4_R is through hepatic growth factor and type 1 tyrosine kinase receptor (c-Met) signaling. As a mediator of the HGF/c-Met pathway, Ang IV stimulates c-Met (Ma et al., 2003; Wright and Harding, 2011), which then attenuates neurodegenerative changes by facilitating the non-NMDA–dependent LTP pathway and increasing dendritic arborization in the hippocampus (Akimoto et al., 2004; Shimamura et al., 2006; Tyndall and Walikonis, 2007). Additionally, the HGF/c-Met system shows cerebroprotective effects by facilitating CBF. All of these functions of the HGF/c-Met system overlap with those of the AngIV/AT4 system (Wright and Harding, 2015). Another manifestation of the learning and memory effects of Ang IV is through regulation of neurotransmitter secretion. In this regard, it has been hypothesized that Ang IV modulates serotonin, dopamine, and acetylcholine release (Lee et al., 2001; Fernando et al., 2008; Gard, 2008). This hypothesis has been supported by studies showing close localization of AT_4_Rs and D2 receptors, correlation of IRAP with cholinergic cell bodies and terminals, and limitation of AngIV-induced cognitive facilitation via blockade of the D2 and D4 dopamine receptors (Chai et al., 2000; Braszko, 2006, 2009; Wright and Harding, 2011).

#### Astrocytes

Astrocytes, microglial cells, and oligodendrocytes are the principal glial cells of the brain. Astrocytes are the most common type of glial cells. Astrocytes have essential roles, such as supporting brain tissue, regulating the chemical content of extracellular space, restricting the spread of neurotransmitter molecules toward unwanted regions by encircling synaptic junctions, and removing many neurotransmitters from the synaptic cleft (Bear et al., 2016; Almad and Maragakis, 2018). These functions make them essential not only for support but also for LTP, thus memory.

Astrocytes are the main angiotensinogen source of the brain. In particular, they have AT_1_R, AT_2_R, and MasR on their cell membranes, mitochondria, and nuclei (Fogarty and Matute, 2001; Garrido-Gil et al., 2013b; Costa-Besada et al., 2018). Although not entirely clear, few studies demonstrate the existence of AT_4_Rs on the astrocytes (Greenland et al., 1996; Holownia and Braszko, 2007). As with neurons, over-activation of AT_1_Rs on astrocytes contribute to oxidative stress, inflammation, cognitive impairment, and cell death while Ang II and Ang (1–7) have protective roles. However, AT_1_Rs on astrocytes differ from those on neurons in that astrocytic AT_1_R activation can affect the permeability of BBB, although it is commonly accepted that overactivation of Ang II/AT_1_R axis increases the permeability of BBB (Biancardi and Stern, 2016; Guo et al., 2019). A study, however, reported counterintuitive results such that astrocyte-derived Ang II restricts BBB permeability with tight junction stabilization, thus diminishing peripheral immune cell entry to the brain (Wosik et al., 2007; Füchtbauer et al., 2010).

#### Microglia

Microglial cells are macrophages that mediate immune and inflammatory responses of the brain (Bear et al., 2016). They can be in two states: resting or activated. In normal conditions, microglia remain in the resting state due to immunosuppressive proteins secreted by neurons. Contrary to their name, resting-state microglia cells actively scan their surrounding environment to detect any abnormalities in brain homeostasis. When they are activated, they can polarize into two distinct substates: proinflammatory/classically activated (M1) and anti-inflammatory/alternatively activated (M2) substates (Labandeira-Garcia et al., 2017). The M1 substate exacerbates neuronal death by secreting pro-inflammatory mediators and free radicals. In contrast, the M2 substrate, being immunoregulatory microglia, promotes brain repair/regeneration, produces growth factors and anti-inflammatory cytokines protecting neurons, and reduces inflammation (Labandeira-Garcia et al., 2017). An inadequate transition from the proinflammatory M1 to immunoregulatory M2 substrate in the presence of any brain lesions may result in the prolonged release of inflammatory cytokines and ROS, which is followed by increased neuroinflammation and neurodegeneration (Kigerl et al., 2009; Heneka et al., 2015; Tang and Le, 2016). It is known that RAS has roles in this immunoregulatory response as it does in the peripheral immune system (Labandeira-Garcia et al., 2017). In particular, RAS affects microglia via AT_1_Rs, AT_2_Rs, and MasRs on mitochondria, nuclei, and cell membranes (Garrido-Gil et al., 2013b; Regenhardt et al., 2013; Costa-Besada et al., 2018). Under non-pathologic conditions, the presence of AT_1_R and AT_2_R are undetectable, unlike the MasR, which can be observed in healthy microglia (Regenhardt et al., 2013; Labandeira-Garcia et al., 2017; Costa-Besada et al., 2018). However, as M1 microglia exerts its pro-inflammatory response, AT_1_Rs and AT_2_Rs are upregulated. In this way, activation of nuclear AT_1_R upregulates itself and leads to a shift toward an M1 phenotype. AT_1_R-mediated activation of the M1 pro-inflammatory response was suggested to be the mechanism that exacerbates cell death and inflammation, ultimately leading to impaired cognition (Bernstein et al., 2014). Similar to what is observed with AT_1_R, activation of nuclear AT_2_R upregulates itself and leads to a shift toward an M2 phenotype. This shift further leads to the production of anti-inflammatory cytokines such as IL-10 and IL-4 and the upregulation of phagocytic receptors that help synaptic clearance (Rodriguez-Pallares et al., 2008; Regenhardt et al., 2013; Biancardi et al., 2015; Fouda et al., 2017). MasR and AT_2_R, both of which have antioxidant and anti-inflammatory properties, also enhance the production of brain-derived neurotrophic factor, known to promote cell survival and synaptic plasticity (Bernstein et al., 2014) and improve cognition (Liu et al., 2016). While AT_2_R expression is usually upregulated alongside AT_1_R as a compensatory mechanism, this relationship was shown to be blunted in aging (Labandeira-Garcia et al., 2017).

#### Oligodendrocytes

Oligodendrocytes are axon-myelinating cells. To our knowledge, they have AT_1_Rs and AT_2_Rs on their cell membrane, which is known to have opposing effects. In particular, for oligodendrocytes, AT_1_Rs lead to demyelination, while AT_2_Rs promote re-myelination to enhance synaptic transmission and improve neuronal communication (Valero-Esquitino et al., 2015; Jackson et al., 2018).

## The Link Between RAS, Oxidative Stress, Neuroinflammation, and Vascular Dysfunction

As reviewed in the previous section, functions of RAS within the brain are not limited to hypertension. Dysregulation of these functions can have detrimental effects on the brain. In this regard, a role for b-RAS has been identified in many different neuropsychiatric disorders, including anxiety, depressive disorder, and alcoholism within the brain (Labandeira-Garcia et al., 2017). Above all, the b-RAS may also lead to chronic neurodegenerative diseases by playing a pivotal role in oxidative stress and neuroinflammation (Barnham et al., 2004). In the following subsections, we will describe the mechanisms through which RAS contributes to oxidative stress and neuroinflammation.

### Oxidative Stress

Although ROS have essential roles in metabolism, cell signaling, and the proper formation of learning and memory processes under physiological conditions (Chandel et al., 2000; Massaad and Klann, 2011; Chandel, 2014), in excessive amounts, they can lead to oxidative stress (Li et al., 2013). ROS are produced as a primary product of NADPH-oxidase (NOX) and secondary products of many other enzymatic processes such as those including xanthine oxidase, cyclooxygenases, uncoupled NOS, and the mitochondrial electron transport chain (De Silva and Faraci, 2013). Among them, membrane NOX complexes and mitochondria are the two main sources of ROS (Babior, 2004). Notably, there is a NOX-derived ROS-mediated cross-talk between them, which can further enhance the production of ROS by mitochondria (Cai, 2005). Furthermore, accumulated amounts of ROS can impair mitochondrial integrity, decrease ATP production, and lead to more mitochondria-derived ROS. It is known that mitochondrial-derived ROS contribute to cellular dysfunction by reaching the cytoplasm, demonstrating additional detrimental consequences.

Reactive oxygen species-associated oxidative stress leads to structural and functional modifications of proteins via oxidation. These modifications can increase the hydrophobicity of some proteins, thus leading to protein aggregation. To restrain cellular toxicity as a consequence of protein aggregation, effective removal of oxidized and damaged proteins is essential. This removal can be performed by either proteasome-mediated protein degradation or the autophagic pathway. However, ROS can also impair the proteasome system, thus leading to reduced protein degradation and accumulation of abnormal proteins such as synuclein, tau, or huntingtin in neurodegenerative diseases (Dröge, 2002; Turrens, 2003). ROS-related inhibition of the proteasome-based protein degradation upregulates autophagy. Although autophagy in response to mild oxidative stress is neuroprotective, its excessive or chronic upregulation promotes cellular death (Dasuri et al., 2013). With these pathways, ROS production and the destructive effects of oxidative stress can be further exacerbated (Gao et al., 2014). Notably, both proteasome-mediated degradation and autophagy demonstrate age-associated dysfunction and the effects of ROS on these processes contributes to the development of age-related neurodegenerative diseases (Grune et al., 2004; Dasuri et al., 2013).

There are seven isoforms of NOX. Among them, NOX1, NOX2, and NOX4 have been identified in the brain (Miller et al., 2007). Overstimulation of the Ang II/AT_1_R axis can activate these brain NOX complexes and generate excessive amounts of ROS (Chan et al., 2005). In this regard, it has been demonstrated that ROS is elevated in the cerebrum with acute and chronic administration of Ang II via activation of NOX2 (Girouard et al., 2006; Chrissobolis et al., 2012). As extracellular Ang II binds to membrane AT_1_R, it activates NOX2, increases intracellular Ca^2+^ levels, and generates intracellular oxidative stress (Wang, 2004). In addition, Ang II is known to be capable of destroying endothelium-dependent vasodilation in the cerebral circulation. However, interestingly, Chrissobolis et al. (2012) showed that these deleterious effects of Ang II did not occur in NOX2 deficient mice treated with Ang II. Oxidative stress also mediates Ang II-induced inward remodeling and hypertrophy in cerebral arterioles that blunt cerebral perfusion in hypertension. Consistent with previous findings, this inward remodeling was also not observed in NOX2 deficient mice (Chan and Baumbach, 2013). Activation of nuclear and mitochondrial AT_1_Rs produce NOX4-derived ROS in neurons by coupling to phosphoinositol-3 kinase and protein kinase C activation (Hongpaisan et al., 2004; Kimura et al., 2005; Abadir et al., 2012; Valenzuela et al., 2016; Villar-Cheda et al., 2017). Importantly, activation of nuclear AT_1_Rs regulates gene expression and triggers several mechanisms that protect cells against oxidative stress (Villar-Cheda et al., 2017). In particular, nuclear RAS increases expression of AT_2_R mRNA and angiotensinogen and activates Ang II/AT_2_R and Ang (1–7)/MasR axes to counteract AT_1_R effects in neurons (Villar-Cheda et al., 2010, 2017; Costa-Besada et al., 2018).

Finally, local Ang II levels impair insulin signaling and contribute to IR by impacting insulin-stimulated increases in insulin receptor substrate1-associated phosphoinositol-3 kinase activity (Folli et al., 1999). In addition to Ang II, hyperglycemia is also known to induce oxidative stress. Therefore, the combined effect of Ang II and hyperglycemia may exacerbate oxidative stress damage in diabetic tissue (Chen et al., 2007; Fukumoto et al., 2008). Separately, abnormalities in brain insulin signaling pathways are associated with cognitive impairment and AD pathology (Arvanitakis et al., 2020).

Besides increasing ROS generation, the RAS can alter the mitochondrial redox balance by diminishing the activity of scavenging enzymes. Many scavengers, including superoxide dismutase (SOD), catalase, and glutathione, have an essential role in reducing oxidative stress in the brain. There are three forms of SOD: cytosolic copper-zinc SOD (CuZnSOD; SOD1), mitochondrial manganese SOD (MnSOD; SOD2), and extracellular CuZnSOD (SOD3). SOD1 and SOD2 are especially crucial for age-related brain disorders (Gao et al., 2014). In transgenic mouse models of Alzheimer’s disease (AD) pathology, the deletion of one allele of SOD2 increased amyloid plaque formation while the deletion of SOD1 increased β-amyloid oligomerization, cognitive impairment, and neuronal dysfunction (Esposito et al., 2006; Murakami et al., 2011). Furthermore, overexpression of SOD was shown to be related to decreased susceptibility to β-amyloid-induced neurotoxicity and ischemic brain injury (Celsi et al., 2004; Chen et al., 2011), yet it has been shown that the activity of antioxidant molecules glutathione, SOD, and catalase decrease with activation of the RAS (Bechara et al., 2005; Rodriguez-Iturbe et al., 2007; Xiong et al., 2010). Consistent with this observation, NO bioavailability and SOD activity increased, and endothelial function improved in humans after treatment with the AT_1_R blocker losartan (Hornig et al., 2001).

ROS production in the brain is high due to its high oxygen consumption, and the oxidative metabolism of neurotransmitters, making the brain extremely vulnerable to additional free radical attacks (Sian et al., 1994; Kumar H. et al., 2012). Therefore, regulation of RAS is particularly essential as it may lead to impaired neurotransmitter release. Within the basal ganglia, cell death and dysfunction increased with AT_1_R upregulation in dopaminergic neurons (Garrido-Gil et al., 2013a; Zawada et al., 2015; Ou et al., 2016). By contrast, in the same type of cells, Ang (1–7)/MasR and Ang II/AT_2_R axes reduced ROS production and mitochondrial respiration (Costa-Besada et al., 2018). Further, it has been shown that upregulated ACE expression reduced acetylcholine release from cholinergic neurons (Barnes et al., 1992; Tota et al., 2012). This RAS-mediated upregulation of oxidative stress leads to the release of pro-inflammatory response exacerbating cell death (Garrido-Gil et al., 2013a; Ou et al., 2016).

### Neuroinflammation

Within the brain, microglial cells are the most significant contributor to NOX-derived ROS (Gao et al., 2012). Among NOX isoforms, NOX2 is the primary source of extracellular ROS in microglia (Gao et al., 2014). NOX-derived ROS also affect intracellular signaling pathways related to microglial activation, proliferation, and release of pro-inflammatory signals (Shacter, 2000; Qin et al., 2004; Shulaev and Oliver, 2006; Gao et al., 2014). Although activation of microglial cells is necessary for removing dead cells and debris, exacerbation of this inflammatory cascade can harm the surrounding neurons and cause the progression of neurodegeneration (Vowinckel et al., 1997).

As discussed in Section “Receptor Functions According to Cell Type,” the Ang II/AT_1_R axis plays a role in the transition of microglia between its activated substates proinflammatory M1 and anti-inflammatory M2. In particular, AT_1_R-induced NOX activation regulates the shift between M1 and M2, with upregulation of the AT_1_R/NOX axis promoting pro-inflammatory and suppressing anti-inflammatory substates. With this signal cascade, microglia further exacerbate ROS production (Choi et al., 2012). This mechanism was also supported by a study showing that AT_1_R antagonists reduce M1 microglia activation and promote M2 microglia polarization (Saavedra, 2017). In microglial cells, AT_1_R-induced NOX activation is mediated by protein kinase C (Joglar et al., 2009). The initial NOX-derived superoxides are amplified by activation of nuclear factor-kβ (NF-kβ) and the RhoA/Rho kinase pathway, which further increased NOX activation, hence yielding ROS production (Borrajo et al., 2014; Rodriguez-Perez et al., 2015). With a feed-forward mechanism, Rho-kinase activation upregulates AT_1_R expression via NF-kβ in microglial cells. Activation of the microglial RhoA/Rho kinase pathway is a significant modulator of the actin cytoskeleton and mediates microglial polarization and neurodegeneration (Labandeira-Garcia et al., 2015, 2017). Moreover, Ang II/AT_1_R axis can increase toll-like receptor 4, which is known to mediate microglial activation and neuroinflammation (Biancardi et al., 2015; Rietdijk et al., 2016). It was shown that Ang II and prorenin promotes the production of ROS and pro-inflammatory cytokines [e.g., interleukin-1 beta (IL-1β) and IL-6] while reducing anti-inflammatory IL-10 levels (Winklewski et al., 2015). As an example, a recent study showed that Ang II administration leads to a pro-inflammatory response in the hippocampus with an increase in hippocampal CD68-positive cells (Takane et al., 2017).

In astrocytes, the Ang II/AT_1_R axis mediates the production of IL-6 and ROS via NF-kβ/ROS pathway (Gowrisankar and Clark, 2016). Also, Ang III can induce signal transducer and activator of transcription 3, which is a crucial signal transduction protein that mediates cell differentiation, proliferation, apoptosis, and inflammation in astrocytes (Costantino and Barlocco, 2008; Kandalam et al., 2015).

There are also inflammation-regulating mechanisms induced through RAS receptors. Neuronal AT_2_R signaling can lead to a downregulated signal transducer and activator of transcription 1 and 3 phosphorylation that can suppress microglia activation (Jackson et al., 2018). AT_4_R was shown to mediate anti-inflammatory effects in a mouse model of AD (Royea et al., 2017). Thus, there is a delicate balance between Ang II/AT_1_R/NOX pro-oxidative and pro-inflammatory axes and Ang II/AT_2_R – Ang (1–7)/MasR antioxidant and anti-inflammatory axis, which determine RAS effects within the brain.

The precise reason for sex differences (premenopausal women vs. men) in the prevalence of cerebrovascular diseases is not yet fully understood. However, an association of Ang II-estrogen was suggested as a possible mechanism due to Ang II’s sexually dimorphic effects on the cerebral vasculature (De Silva and Faraci, 2013). Consistent with this suggestion, it was shown that activation of microglial estrogen receptor B suppresses Ang II-induced increase in levels of several significant mediators (e.g., IL-1β and rho kinase) of the microglial inflammatory response (Rodriguez-Perez et al., 2015).

### Vascular Dysfunction

Renin–angiotensin system can affect CBF through several mechanisms. The first such mechanism acts through Ang II-induced hypertension, which is characterized by many forms of alterations in the cerebral vasculature, including pathological remodeling of arteries, vasoconstriction, diminished cerebrovascular autoregulation, cerebrovascular inflammation, and decreased vascular compliance (Faraci and Heistad, 1990). A consequence of this RAS/hypertension-based mechanism is the development of an impaired response to reduced cerebral perfusion, which may render the brain vulnerable to ischemia and cellular injuries (Saavedra, 2017).

Renin–angiotensin system can also cause vascular dysfunction independent of hypertension. Based on its pro-oxidative and pro-inflammatory properties, it can cause vasoconstriction, endothelial damage, BBB breakdown, and disruption of neurovascular coupling (Ahmed et al., 2019). Impairments of neurovascular coupling, defining the alterations in CBF according to local neural activity, was shown to be associated with Ang II independent of hypertension based on evidence in phenylephrine-induced hypertensive mice showing no impairment in their neurovascular coupling (Kazama et al., 2003; Capone et al., 2011). Similarly, the detrimental effects of the Ang II/AT_1_R axis on BBB permeability have not been seen in a deoxycorticosterone acetate-induced hypertension state (Rodrigues and Granger, 2012). Ang II can induce vasoconstriction via smooth muscle- and endothelium-dependent mechanisms. First of all, smooth muscle contraction generally occurs via AT_1_R-mediated activation of phospholipase C, resulting in inositol 1,4,5-triphosphate and diacylglycerol production. These products mediate two distinct pathways, which commonly result in smooth muscle contraction via activation of several protein kinases, such as myosin light chain kinase and Rho-kinase (Hilgers and Webb, 2005). Ang II-induced constriction of basilar arteries was destroyed by Rho-kinase inhibitor Y-27632 (Faraci et al., 2006). Another path to vasoconstriction is endothelium-dependent constriction via cyclooxygenase- and/or superoxide-dependent mechanisms mediated by AT_1_R in cerebral endothelium (De Silva et al., 2009). Finally, the Ang II/AT_1_R axis causes an alteration in endothelium-dependent vasodilation by decreasing the bioavailability of NO (Intengan and Schiffrin, 2001).

## Age- and Disease-Related Changes in the RAS

The pathophysiologies reviewed in the last section generally develop in the elderly. In this section, we discuss their connection with aging and how they lead to frailty and neurodegenerative diseases.

### RAS, Aging, and Frailty

Aging and RAS are interrelated. Age-associated unbalanced activation of RAS influences the aging phenotype. RAS component levels changing with age accelerate cellular senescence and age-related tissue/organ dysfunction, thus leading to chronic age-related diseases through various mechanisms including oxidative stress, inflammation, apoptosis, and vascular changes. Several studies investigated the relationship between RAS, longevity, and neurodegenerative diseases. The relationship between RAS and longevity was first shown in mice by demonstrating that disruption of the AT_1_R promotes longevity via attenuation of oxidative stress and pro-survival gene induction (Benigni et al., 2009). Benigni et al. (2013) also showed the association between prolonged life span and reduced AT_1_R protein in humans with AT_1_R gene variation. Furthermore, it has been shown in multiple studies that Ang II mediates premature senescence (Kunieda et al., 2006; Tsai et al., 2016).

Changes in RAS components with age are differently regulated in circulating and local RAS (Abadir, 2011). While circulating RAS components have lower levels in older animals, perhaps, due to age-related increase in blood pressure (Diz, 2008), AT_1_R levels in local RAS are found to be upregulated (Kobori et al., 2007; Abadir, 2011; Abadir et al., 2011; Rodriguez-Perez et al., 2019). Previous work investigating changes in AT_1_R and AT_2_R level with age showed that mitochondrial and nuclear AT_1_R levels increased significantly with age (Gwathmey et al., 2010; Abadir et al., 2011) as opposed to decreased AT_2_R expression (Millan et al., 1991). Furthermore, the maximal expression of AT_2_R was found in developing fetal tissues, followed by an immediate reduction after birth and maintenance of relatively lower levels in adulthood (Carey and Siragy, 2003). This was also confirmed by increased AT_1_R/AT_2_R expression ratios in aged animals (Garcia-Garrote et al., 2019). This was attributed to age-associated changes in the distribution of Ang II receptors, with nuclear AT_1_Rs becoming the most common (85%) in older ages as opposed to nuclear AT_2_Rs being the most common (80%) at young ages (Gwathmey et al., 2010). These distributional alterations were also shown in mitochondria of aged mice (Abadir et al., 2011). However, the same study showed that *in vivo* administration of AT_1_R blocker losartan for 20 weeks increased the number of mitochondrial AT_2_Rs and improved bioenergetics of the aging mitochondria without affecting the expression of mitochondrial AT_1_Rs (Abadir et al., 2011).

The b-RAS distribution also shows age-related differences. In a recent study, a progressive decrease in the expression of AT_2_Rs with aging was shown in the SN (Rodriguez-Perez et al., 2019). Notably, in the same study, several changes in external appearance, spontaneous motor behavior, RAS components, and pro-oxidative and pro-inflammatory markers were found in young AT_2_R KO mice as being similar to those observed in aged wild type mice. Regarding RAS changes, elevated Ang II and AT_1_R levels and decreased mRNA/protein expression of other protective RAS receptors were observed both in aged wild type and AT_2_R-KO mice (Rodriguez-Perez et al., 2019). Another study using AT_2_R-KO mice found increased AT_1_R expression in the brain ventricular–subventricular zone accompanied by a marked decrease in proliferation and generation of neuroblasts. This decrease was inhibited by treatment with the AT_1_R antagonist candesartan. Further, increased proliferation and generation of neuroblasts were shown in wild type mice with the administration of AT_2_R agonist C21 (Garcia-Garrote et al., 2019). Similarly, it was shown that AT_1_R blockade can enhance hippocampal neurogenesis in hypertensive state (Bhat et al., 2018; Drews et al., 2019). Interestingly, neurogenesis was enhanced in hippocampal dentate gyrus of adult rodents exposed to short-term heat as a result of activation of AT_1_R due to increase in Ang II (Koyama et al., 2018). This finding was in contrast to the aforementioned literature and might be because of the transient increase of Ang II with short-term application of the heat. However, further research is needed for a more definitive explanation. In another study about the importance of RAS on neurogenesis showed that permanent depletion of ACE2 impaired the effect of running on neurogenesis in the adult hippocampus (Klempin et al., 2018). Finally, similar observations were made for MasRs (Gwathmey et al., 2010) such that the protective Ang1–7/Mas axis expression decreased in aged rats similar to AT_2_R (Villar-Cheda et al., 2010; Costa-Besada et al., 2018).

Mitochondrial dysfunction has a pivotal role in cell aging. The distributional changes in RAS receptor levels in mitochondria with aging in favor of AT_1_Rs elevates mitochondrial ROS levels. This can diminish mitochondrial integrity and function, leading to decreased ATP generation and further production of ROS and peroxynitrite, a cytotoxic anion that inhibits mitochondrial electron transport (Sastre et al., 2000; Abadir et al., 2012). ROS have been proposed as the leading molecular factor in the aging process (Harman, 1956). One cellular aging mechanism is one in which increased mitochondrial ROS levels lead to oxidation of mitochondrial protein/lipid and DNA mutations whose accumulations are linked to senescence and apoptosis (Abadir et al., 2012; Vajapey et al., 2014). Aggregation of modified proteins due to enhanced RAS activity could be the mechanism of accelerated premature aging in AD (Cooper et al., 2018).

Consistent with the detrimental effects of AT_1_Rs on mitochondria, it was shown that deletion of AT_1_Rs resulted in a marked prolongation of life span in mice by upregulating multiple mitochondrial and prosurvival genes, nicotinamide phosphoribosyltransferase (Nampt) and sirtuin 3 (SIRT3) in the kidney (Benigni et al., 2009). Similarly, it was shown within the brain that overactivation of the Ang II/AT_1_R/NOX axis leads to increased cell vulnerability to oxidative stress by decreasing levels of SIRT3, which would typically suppress pro-oxidative RAS axis with a feed-forward mechanism (Diaz-Ruiz et al., 2020). The same study also showed that these effects could be counteracted by treatment with AT1 antagonists in aged rats (Diaz-Ruiz et al., 2020). On the other hand, SIRT1 and Ang II/AT_1_R axis, both of which have roles in neuroinflammation, oxidative stress, and aging-related cell degeneration, also regulate each other, and this regulation is impaired in aged animals (Diaz-Ruiz et al., 2015). Further, lower SIRT1 in brain is associated with more brain tau pathology (Julien et al., 2009). In addition, glial-derived Ang II appears to participate in age-related impairments of autonomic function such that glial angiotensinogen deficiency prevents these impairments (Diz, 2008). The intracellular Ang II/AT_1_R/NOX axis generates superoxide anions that promote the uncoupling of endothelial NOS, which in turn reduces NO availability and mitochondrial NOS activity with aging (Valdez et al., 2004). Finally, the Ang II/AT_1_R axis accelerates cellular senescence by inducing telomere shortening and cell cycle arrest, which can be reversed by losartan (Feng et al., 2011; Conti et al., 2012). Similar to Ang II, agonistic angiotensin II type 1 receptor autoantibodies can activate the AT_1_R (Herse and LaMarca, 2013), which is consistent with the finding that higher levels of autoantibodies are associated with higher levels of inflammatory cytokines, weaker grip strength, slower walking speed, and increased risk for frailty (Abadir et al., 2017).

The compensatory mechanism of the b-RAS, which decreases oxidative stress and neuroinflammation, becomes less effective with age due to reduced AT_2_R with age (Villar-Cheda et al., 2010, 2012; Lu et al., 2015). In aged animals, this decrease in AT_2_R expression was shown to lead to a further increase in the pro-oxidative, pro-inflammatory effects and neuron vulnerability induced by activation of upregulated AT_1_Rs (Labandeira-Garcia et al., 2017). As an example of neuronal vulnerability, neurotoxins lead to more dopaminergic neuron loss in aged compared to younger animals (McCormack et al., 2004).

The imbalance developing with age between pro-inflammatory and protective arms of RAS leads to inflammaging, which refers to aging-related pro-inflammatory changes observed in several tissues (Abadir, 2011; Labandeira-Garcia et al., 2017). This is associated with the pathogenesis of common and disabling diseases of older age, functional decline, frailty, and increased mortality (Abadir, 2011). Consistent with this, increased NOX activity due to RAS was seen in age-related diseases such as diabetes and atherosclerosis (Griendling et al., 2000; Mehta and Griendling, 2007). Aging and neuroinflammation in brain tissue together lead to exacerbated responses to lesions and neurodegeneration, hence being major risk factors for neurodegenerative diseases such as AD and Parkinson’s disease (PD) (Collier et al., 2007; Labandeira-Garcia et al., 2017).

Lifelong accumulation of neuropathologies occurs in neurodegenerative diseases prior to clinical diagnosis (Braak and Braak, 1991; Bennett et al., 2012). Although neuropathologies are responsible, in large part, for the onset of dementia, they only account for a fraction of cognitive decline (Boyle et al., 2013, 2018, 2019). In this regard, frailty may be a critical determinant of the cognitive impairment in both AD and stroke (Bennett et al., 2012; Taylor-Rowan et al., 2019). A strong interaction between frailty and pathologies was shown in AD such that high frailty renders subjects more vulnerable to pathologies with both more frequent development of the disease and worse cognitive decline. By contrast, people with low frailty are more resilient to neuropathologies (Wallace et al., 2019).

Frailty has also been shown to be an independent factor determining both the prevalence of stroke and post-stroke mortality rates (Palmer et al., 2019; Evans et al., 2020). The last decade has witnessed a considerable increase in research on frailty. Frailty is strongly influenced by multiple factors, including inflammation, oxidant state, vascular regulations, and mitochondria dysfunctions, and apoptosis. RAS plays a broad and essential role in the regulation of these factors, and it is closely associated with both physical and cognitive frailty phenotypes. Given the availability of RAS-acting drugs and the canonical role of RAS in both frailty and pathological mechanisms of age-related neurodegenerative diseases, which is discussed in the subsequent sections, RAS deserves further research as a potential therapeutic agent in age-related diseases.

### Alzheimer’s Disease

Alzheimer’s disease is a complicated neurodegenerative disease characterized by progressive neuronal losses and cognitive impairment. Based on the brain autopsies of 83 patients with and without dementia, Braak and Braak (1991) described the pathologic characteristics of the disease as degeneration of specific nerve cells, presence of neuritic plaques, and neurofibrillary tangles (NFT). The most prominent pathological changes were found to be the extracellular amyloid plaques and intraneuronal NFT accumulations, which had started to take place years before the appearance of clinical symptoms (Braak and Braak, 1991; Mcgeer and Mcgeer, 1995). Although it is possible that these accumulations are not causal in AD pathogenesis, they characterize AD as a unique neurodegenerative disease among different diseases that can lead to dementia. However, classical diagnostic approach focused solely on clinical syndromes of AD regardless of these unique AD neuropathologies. This has resulted in misdiagnosis and limited our understanding of AD on a biological basis. Thus, recently, a purely biological definition of AD was introduced based on *in vivo* identification and postmortem examination of distinctive AD neuropathologies (Jack et al., 2018). This new research framework recommends referring to the clinical symptoms of AD without neuropathologic verification as Alzheimer’s clinical syndrome instead of what has been called ‘probable or possible AD’ according to the traditional approach (Jack et al., 2018). Various RAS-related mechanisms have been suggested as contributors to its pathogeneses. The b-RAS is involved with processes of learning and memory, neuronal differentiation, and nerve regeneration via several mechanisms, including oxidative stress, neuroinflammation, and vasculopathy, as discussed in the previous sections. The relation of AD and other pathologies to cognitive decline follow complex patterns (Boyle et al., 2017; Wilson et al., 2019). A few years before death the rate of cognitive decline increases markedly due to terminal decline (Wilson et al., 2012). Several predisposing factors, such as genetics, age, and possibly environmental toxins, contribute to the development of initial lesions like senile plaques and NFTs. These products lead to an inflammatory response, microglial activation, and cytokine release, which then accelerates neuronal dysfunction and cognitive decline (Mcgeer and Mcgeer, 1995). In this regard, inflammation was thought to contribute to the progressive neuropathology of AD (McGeer and McGeer, 2013). As discussed in Section “Receptor Functions According to Cell Type,” the Ang II/AT_1_R axis of the b-RAS is an important contributor to neuroinflammation by enhancing microglial activation and polarization. Therefore, prolonged and unresolved inflammation harm neurons and synapses, which results in chronic dysregulation of glial cells followed by chronic deterioration in the brain structure and function (Denver and McClean, 2018). Furthermore, a recent study showed that higher Ang II levels are associated with smaller total gray matter, hippocampal, rostral middle frontal, and supramarginal parietal volumes which are related to cognitive domains that may decline in preclinical AD (Yasar et al., 2020).

A complementary explanation suggests the relation among increased oxidative stress, mitochondrial impairment, and alterations in the antioxidant system as a contributor to AD (Padurariu et al., 2013). As described in Section “Prelude to the Renin–Angiotensin System Signaling and Functions,” over activity of the Ang II/AT_1_R axis causes increased ROS and oxidative stress. First, oxidative stress causes damage by lipid peroxidation of the mitochondrial membrane as well as oxidation of structural and enzymatic proteins and nucleic acids. Increased oxidation of mitochondrial DNA impairs mitochondrial integrity and decreases ATP production, thus potentially resulting in mitochondrial dysfunction (Mecocci et al., 2018). As mitochondrial function declines, it leads to cellular alterations observed in AD, such as amyloid-β (Aβ) production, tau phosphorylation, synaptic degeneration, and oxidative stress (Swerdlow, 2011, 2012). Secondly, oxidative stress-associated modifications of the proteins can result in aggregation of Aβ and phosphorylation of tau protein, which could induce a vicious cycle of pathogenesis in AD (Kim et al., 2015). The amount of antioxidants is also an essential factor determining the extent of oxidative damage in the pathogenesis of AD (Mecocci et al., 2018). Their importance is supported by data showing neuroprotective effects of some anti-oxidants such as γ-tocopherol and lycopene (Yin et al., 2014; Morris et al., 2015).

Amyloid-β accumulation is another important aspect of AD pathogenesis. Ang II enhances the γ-secretase activity and Aβ production (Zhu et al., 2011). Aβ production leads to AT_2_R oligomerization, which is associated with enhanced neurodegeneration (AbdAlla et al., 2009). In addition to the Ang II/AT_1_R axis, ACE has also been investigated for its possible role in Aβ degradation (Miners et al., 2011). Although ACE was shown to degrade Aβ peptide (Hemming and Selkoe, 2005), it can also degrade neprilysin, an Aβ-degrading enzyme that may contribute to Aβ aggregation (Carson and Turner, 2002). Moreover, it has been shown in brain tissue autopsy of AD patients that ACE levels are elevated in the hippocampus, frontal cortex, and caudate nucleus regardless of hypertension and that these levels have been reported to correlate with AD pathology (Miners et al., 2008, 2009). Similarly, it was shown in a recent study that cerebrospinal fluid (CSF) ACE activity was elevated in AD (Kehoe et al., 2019). Furthermore, the same study showed that RAS overactivity is correlated with CSF markers of capillary damage in AD, including elevated CSF soluble platelet-derived growth factor receptor β indicating pericyte damage and elevated CSF albumin indicating BBB breakdown.

Vascular disease may also contribute to the pathogenesis of AD. This is supported by the fact that many risk factors for vascular disease such as hypertension and diabetes are associated with Alzheimer’s dementia (de La Torre, 2004). These relationships are complex and the association is likely due in part to mixed pathologies (Abner et al., 2016; Pruzin et al., 2017; Arvanitakis et al., 2018). The progressive degeneration of brain capillaries, such as thickened basement membrane, cerebral atrophy, reduced vessel elasticity, or genetic predisposition, disturbs the blood flow to the brain (de la Torre and Mussivand, 1993). The hypothesis proposes the impaired blood flow in conjunction with neuroinflammation as the central reason for Aβ aggregates and neuronal damage in the AD (de la Torre, 2002). RAS can modulate both of these underlying factors. In this regard, Ang II/AT_1_R axis causes vasoconstriction of the cerebral vessels, vascular remodeling, impaired cerebrovascular autoregulation, and endothelial dysfunction (Iadecola and Davisson, 2008; Pires et al., 2013). As mentioned so far, Ang II/AT_1_R axis has pro-inflammatory and pro-oxidant effects that can damage the BBB, increase its permeability, and reduce CBF, thus contributing to the pathogenesis of the AD (Miners et al., 2011). Consistent with this, blockade of AT_1_R and activation of AT_2_R reverse the hypertension-induced cerebrovascular dysfunction and improve the barrier function of endothelial cells and diabetes-associated cerebral endothelial dysfunction (Alhusban et al., 2013; Gallego-Delgado et al., 2016; Fouda et al., 2019).

Interestingly, there is a relationship between AD and diabetes mellitus type 2, which are both age-associated diseases (Denver and McClean, 2018). IR increases the risk of developing cognitive decline, and IR has been shown in postmortem brain tissue of AD patients without diabetes (Talbot et al., 2012; Arvanitakis et al., 2020). Also, impaired neuronal insulin signaling was demonstrated in the AD brain (Denver and McClean, 2018). In the brain, normally functioning insulin signaling is very important for proliferation, differentiation, and neurite growth. Insulin plays an essential role in learning and memory (Blázquez et al., 2014). In this regard, it was shown in rats in a water-maze task that long-term fructose-drinking causes IR, impaired insulin signaling, oxidative stress, neuroinflammation, the down-regulated activity of the cholinergic system, cognitive decline, impairments of spatial memory and learning (Yin et al., 2014). IR also causes endothelial dysfunction, which is important for AD development (Aronis and Mantzoros, 2012). Therefore, another impact of RAS in the aspect of AD might be impaired insulin signaling and its contribution to IR (Folli et al., 1999) through mechanisms mentioned in Sections “Neurons” and “Oxidative Stress.” Interestingly, a significant increase in intracellular ACE was shown in high glucose conditions despite no change in extracellular ACE under the same circumstances (Cristovam et al., 2008).

Finally, b-RAS has important learning and memory-related effects (Ho and Nation, 2018). Among these effects, the overactivation of the Ang II/AT_1_R axis is known to decrease acetylcholine release (Barnes et al., 1992; Tota et al., 2013). This might complement the role of cholinergic dysfunction in AD, proposing the loss of acetylcholine in the central nervous system as an important determinant of the cognitive decline in AD (Bartus et al., 1982; Kehoe, 2018). It was also shown that Ang II injection inhibits LTP in the hippocampus. On the other hand, the protective arm of the RAS has memory-enhancing effects. AT_2_R activation through Ang II/III initiate cellular proliferation and differentiation accompanied by regenerative processes. Ang (1–7)/MasR axis enhances the release of NO and facilitates LTP, thus resulting in improved memory (Wright and Harding, 2019). Ang IV enhances dopamine release in the striatum and acetylcholine release in the hippocampus, thus facilitating LTP and neuroprotection (Lee et al., 2001; Lew et al., 2003; Stragier et al., 2004; Davis et al., 2006). Ang IV increases concentrations of cognition-enhancing peptides such as vasopressin and oxytocin by inhibiting aminopeptidase activity of ATR4 (Tomizawa et al., 2003; Bielsky et al., 2005). Ang IV can stimulate angiogenesis and enhance NMDA currents and synaptic plasticity in the hippocampus by binding the c-Met receptor (Akimoto et al., 2004). Ang IV could increase CBF without significant changes in systemic blood pressure (Wright and Harding, 2019).

Renin–angiotensin system lies at the intersection of pathologies of AD through many mechanisms mentioned so far, thus being a potentially critical component of AD pathogenesis. In the light of this, angiotensin hypothesis was suggested for further research (Kehoe, 2018). Finally, the ready availability of RAS-modifying drugs makes RAS more attractive as those drugs can be repurposed for the prevention and treatment of AD.

### Parkinson’s Disease

Parkinson’s disease is a common neurodegenerative disease affecting more than 6 million individuals globally according to a recent study (Dorsey et al., 2018). It is characterized by dysregulation of the dopaminergic pathways and neuronal death as with other neurodegenerative diseases. Aging and neuroinflammation are two critical factors that have roles in the development and progression of PD. Brain RAS modulates both of these factors in SN (Valenzuela et al., 2010; Garrido-Gil et al., 2013b). The relationship between RAS and PD was first defined by Allen et al. (1992). It has been shown in several studies that b-RAS has a significant role in the progression of dopaminergic neuron degeneration in PD models (Grammatopoulos et al., 2007; Zawada et al., 2011; Sonsalla et al., 2013; Labandeira-Garcia et al., 2017). Moreover, increased CSF ACE activity in PD patients and an association between genetic polymorphism of the ACE gene and PD were also shown (Almeida-Santos et al., 2017).

The Ang II/AT_1_R axis acting presynaptically in the SN and striatum enhances dopamine release (Mendelsohn et al., 1993; Brown et al., 1996). However, overactivation of this axis surprisingly shows a reverse effect, contributing to the loss of dopaminergic neurons and progression of neurodegeneration in PD models through oxidative stress induced by NOX complex activation and enhanced neuroinflammation mostly via microglia activation (Costa-Besada et al., 2018). Interestingly, Ang II/AT_1_R axis did not lead to dopaminergic neuronal death in the absence of microglia (Joglar et al., 2009). These findings show the importance of microglia in PD. Finally, in PD, low dopamine levels further increase neuroinflammation and neurodegeneration via the upregulation of the AT_1_R/NADPH-oxidase axis (Rodriguez-Perez et al., 2019).

Renin–angiotensin system also has protective roles against the progression of PD. The Ang II/AT_2_R axis can lead to actions opposing those of the Ang II/AT_1_R axis in SN (Costa-Besada et al., 2018). Besides, Ang IV stimulates c-Met, and activation of the HGF/c-Met pathway inhibits the dopaminergic neuron loss in the SN in rats. Furthermore, maintenance of AT_1_R and AT_2_R balance via external modulations with AT_1_R blockage or hormonal replacement therapy-based AT_2_R upregulation was shown to be beneficial in PD (Farag et al., 2017). In addition, it has been shown that RAS blockade can reduce motor and non-motor symptoms of PD and neuronal damage (Almeida-Santos et al., 2017). For example, the AT_1_R antagonist candesartan decreased the expression of nigral proinflammatory cytokines and dopaminergic cell vulnerability to neurotoxins in aged rats (Villar-Cheda et al., 2012). Finally, a similar modulation was obtained with estrogen replacement therapy, which led to a reduced nigral RAS and oxidative stress in young surgically menopausal rats than in aged menopausal rats. However, a remarkable reduction in dopaminergic neuron loss in both groups of menopausal rats was seen with the AT_1_R antagonist candesartan (Rodriguez-Perez et al., 2012).

### Vascular Cognitive Impairment

Cerebrovascular disease is common and often followed by brain dysfunction, further leading to cognitive loss, which is called vascular cognitive impairment (Gorelick et al., 2011; Arvanitakis et al., 2016). Cognitive impairment is seen in nearly 30–40% of stroke survivors and progresses slowly even after a single-stroke lesion (Wiesmann et al., 2013; Levine et al., 2015). Two major risk factors of both stroke and the subsequent cognitive impairment are aging and hypertension, both of which affect CBF through vascular dysfunctionalities discussed in Section “Vascular Dysfunction” (Ivan et al., 2004; Ahmed et al., 2019). Diminished CBF leads to hypoperfusion and hypoxia by creating a pro-oxidative and pro-inflammatory environment in the brain, which eventually results in neuronal death, thus contributing to the development of cognitive impairments (Ahmed et al., 2019).

Because of its pro-oxidative and pro-inflammatory properties, AT_1_R overactivity plays an essential role in vascular cognitive impairment (Arroja et al., 2016). In this regard, AT_1_R overactivity promotes vasoconstriction, reduces CBF, and increases oxidative stress, inflammation, and vulnerability to ischemia (Labandeira-Garcia et al., 2014). It was also shown that AT_1_R-induced astrocyte senescence exacerbates cerebral ischemic injury (Liu et al., 2011). By contrast, the protective arm of RAS counteracts these effects. Six hours after ischemic injury in rodents, the MasR was found to be upregulated in the peri-infarct cortex (Arroja et al., 2016). Another study, reporting a similar overexpression of ACE2 and MasR in ischemic tissues, suggested that the Ang (1–7)/MasR axis potentially plays a pivotal role in the regulation of acute neuron injury in ischemic cerebrovascular diseases (Lu et al., 2013). MasR activation and ACE2 overexpression reduced both inducible NOS and the production of pro-inflammatory cytokines in the peri-infarct cortex (Bedecs et al., 1997; Xia and Lazartigues, 2008; Gwathmey et al., 2009; Arroja et al., 2016). Because of its anti-oxidative, anti-inflammatory, and neuroprotective properties, the protective arm of RAS has been studied as a therapeutic option for vascular cognitive impairment. In this regard, the Ang (1–7)/MasR axis was shown to have proangiogenic properties; administration of Ang (1–7) for 4 weeks promoted brain angiogenesis (Jiang et al., 2014). MasR expression was found in large amounts in hippocampus, perirhinal cortex, and vascular endothelial cells, and it has been shown that Ang (1–7)/MasR axis facilitate LTP (Hay et al., 2017). Glycosylated Ang (1–7)/MasR agonist improved object recognition and spatial memory impairment and reduced ROS and inflammation in mouse models of VCI and dementia (Hay et al., 2019). Interestingly, in a study comparing wild-type mice, MasR KO mice, AT_2_R KO mice, and AT_2_R/MasR double KO mice, it was found that cognitive status was unchanged in MasR KO mice despite decreased cerebral blood flow after bilateral carotid artery stenosis (Higaki et al., 2018).

Internal carotid artery administration of increasing doses of Ang IV significantly decreased cerebral infarct size in rats 24 h following embolic stroke, possibly due to Ang IV-facilitated the redistribution of blood flow to ischemic areas within a few minutes as indicated by cerebral arteriography (Faure et al., 2006). Furthermore, it was shown that C21, an AT_2_R agonist, prevented the development of cognitive impairment after stroke in aged animals and animal models of vascular dementia (Iwanami et al., 2015; Ahmed et al., 2019). The use of ARBs showed both vascular and neuroprotective effects after ischemic stroke and preserved cognitive function in aged animals with chronic cerebral hypoperfusion (Alhusban et al., 2015; Ahmed et al., 2018). Lastly, the TROPHY (TRial Of Preventing Hypertension) study also showed protective effects of valsartan against ischemic brain injury after middle cerebral artery occlusion in mice given non-hypotensive doses (Li et al., 2008).

## Renin-Angiotensin System-Acting Drugs

As of today, an effective risk reduction, prevention, or treatment strategy is not available for AD. Over 20 years, two categories of drugs have been used for the treatment of AD. One of them is cholinesterase inhibitors that extend the half-life of acetylcholine, whereas the other is memantine, an NMDA receptor antagonist, that limits glutamate excitotoxicity and neuronal damage (Wright and Harding, 2019). With the discovery of b-RAS and its multidimensional effects on the nervous system beyond its well-known hypertensive effect, RAS-acting drugs have been considered as a potential preventive and therapeutic intervention for neurodegenerative diseases. There are three types of RAS-acting drugs: ARBs, ACEIs, and direct renin inhibitors (DRI), each of which act at different steps of the RAS process. While ARBs (e.g., losartan, valsartan, telmisartan, and candesartan) block binding of Ang II to the AT_1_R, ACEIs (e.g., captopril, enalapril, lisinopril, and perindopril) block the hydrolysis of Ang I to Ang II. The blockade of AT_1_R results in increased Ang II levels and consequently increases stimulation of AT_2_R. In contrast, ACEI causes lower Ang II levels and consequently reduces stimulation of both AT_1_R and AT_2_R (Düsing, 2016). It was hypothesized that ARBs have potential advantages over ACEIs in the prevention of cognitive impairment because of more specific concomitant blocking of harmful effects while possibly enhancing beneficial effects such as vasodilatation and endothelial modulation (Fournier et al., 2004; Anderson et al., 2011). DRIs directly inhibit generation of Ang I from angiotensinogen, acting upstream of both ARBs and ACEIs (Riccioni, 2013). RAS inhibitors can reduce Aβ deposition and its consequences, and also suppress inflammation, oxidative stress, vascular damage/ischemia, and increase acetylcholine release and glutamate uptake (Gebre et al., 2018; Lebouvier et al., 2020). Especially, ARBs can prevent impairment of the BBB and reduce infiltration of inflammatory mediators observed in many neurodegenerative disease such as AD (Gebre et al., 2018). A summary of studies examining RAS-acting drugs in cognition and dementia is given in Table 1.

**TABLE 1:** Summary of research studies evaluating the effects of RAS-acting agents on cognition and dementia.

**Author/year**	**Study design**	**Number of patients**	**Mean age (years)**	**Follow-up (years)**	**Treatment**	**Disease**	**Results**
Tzourio et al., 2003	Double-blind RCT	6,105	64	3.9	ACEI	Dementia	Active treatment (perindopril with or without indapamide) was associated with reduced risk of dementia (relative risk reduction, 12% [95% CI, −8% to 28%]; *P* = 0.2). Cognitive decline was seen in 9.1% of the actively treated group and 11.0% of the placebo group (risk reduction, 19% [95% CI, 4% to 32%]; *p* = 0.01).
Ohrui et al., 2004a	Cohort study	4,124	69	8	ACEI	AD	Central-acting captopril and perindopril were associated with a significantly lower incidence of AD than the use of those that cannot inhibit brain ACE (imidapril or enalapril) (odds ratio = 0.25, 95% CI = 0.08–0.75; *p* = 0.014).
Ohrui et al., 2004b	RCT	162	76	1	ACEI	AD	The mean 1-year decline in MMSE scores in the participants of brain-penetrating ACEIs (perindopril or captopril) was lower than those in the participants of non-brain-penetrating ACEIs (imidapril or enalapril) and CCBs (*p* < 0.001).
Khachaturian et al., 2006	Cohort study	3,217	74.9	3	ACEI	AD	In the analyses of AD risk among different types of antihypertensive medications, ACEIs (HR, 1.13; 95% CI, 0.60–1.98) and CCBs (HR, 0.86; 95% CI, 0.45–1.53) showed no impact on AD risk.
Li et al., 2010	Prospective cohort analysis	819,491	74	4	ARB/ACEI	AD	A significant reduction in the occurrence of AD was identified with ARBs compared to lisinopril (HR 0.81, 95% CI 0.68–0.96, *P* = 0.016).
Yasar et al., 2013	*Post hoc* analysis of RCT	1,928	78.6	6.1	ARB/ACEI	AD	HR for AD occurrence among participants with normal cognition was found 0.51 in diuretic (95% CI 0.31–0.82), 0.31 in ARB (95% CI 0.14–0.68), 0.50 in ACEI (95% CI 0.29–0.83), 0.62 in CCB (95% CI 0.35–1.09), and 0.58 in BB (95% CI 0.36–0.93) users.
Barthold et al., 2020	Retrospective cohort study	694,672	77.3	7	ARB/ACEI	AD	The annual AD and related dementias incidence rate was found 2.07% among persons using a RAS-acting antihypertensive and any statin, and 2.64% among persons using a non-RAS-acting antihypertensive and any statin. ACEI + pravastatin OR = 0.942 (CI: 0.899–0.986, *p* = 0.011), ACEI + rosuvastatin OR = 0.841 (CI: 0.794–0.892, *p* < 0.001), ARB + pravastatin OR = 0.794 (CI: 0.748–0.843, *p* < 0.001), ARB + rosuvastatin OR = 0.818 (CI: 0.765–0.874, *p* < 0.001).

*ACEI, angiotensin-converting enzyme inhibitor; ARB, angiotensin receptor blocker; CCB, calcium-canal blocker; BB, beta-blocker; AD, Alzheimer’s disease; RCT, randomized controlled trial; HR, hazard ratio; CI, confidence interval; OR, odds ratio*.

### Angiotensin Receptor Blockers

#### Studies Showing Positive Effects on Cognition and Neural Protection

Early studies investigated cognitive effects of ARBs in animals. It was shown that pretreatment with losartan reduced chronic ethanol-induced cognitive deficits (Tracy et al., 1997) and improved both spatial and short-term working memory in animals (Raghavendra et al., 1998; Royea et al., 2020). It was shown that telmisartan has a long term anti-inflammatory effect by modulating microglia *in vitro* and *in vivo* (Torika et al., 2016). Chronic intranasal administration of losartan decreased plaque number in an AD mouse model (Danielyan et al., 2010) and also attenuated Ang II-induced cognitive impairment and tau phosphorylation (Tian et al., 2012). Candesartan was found to be more effective than perindopril in blunting the neuroinflammation in both astroglial and microglial cells of the rat brain (Bhat et al., 2016). An *in vitro* study screening 55 commonly prescribed antihypertensive drugs with respect to their anti-Aβ properties found that only valsartan and losartan are capable of both lowering Aβ and decreasing oligomerization of Aβ peptides in primary neuronal cultures, while candesartan was effective on oligomerization only (Wang et al., 2007; Zhao et al., 2009). Preventive treatment with valsartan significantly reduced brain Aβ deposits and Aβ-mediated cognitive deterioration, independently of blood-pressure lowering in AD mice model (Wang et al., 2007). Interestingly, it was shown that olmesartan and losartan protect cognitive function and cerebrovascular activity independently from blood pressure changes through decreasing oxidative stress in brain microvessels without decreasing Aβ levels (Takeda et al., 2009; Ongali et al., 2014). Some ARBs especially telmisartan were found to be partial agonists of peroxisome proliferator-activated receptor-γ (PPAR-γ) which is a target in AD with its anti-inflammatory, anti-amyloidogenic and insulin-sensitizing effects. In this regard, it was hypothesized that beneficial effects of telmisartan are attributed not only to its AT_1_R antagonist properties but also to the activation of PPAR-γ (Heneka et al., 2007; Torika et al., 2016; Lebouvier et al., 2020). In line with this, it was shown that diabetes-induced increases in BBB permeability was attenuated by telmisartan through PPAR-γ activation to improve diabetes-induced cognitive decline (Min et al., 2012).

One of the earliest human studies was a double-blind randomized control trial which compares losartan and hydrochlorothiazide in 69 elderly patients. It was shown that losartan has a positive effect not only on blood pressure but also on impaired cognitive function, reversing even minimal cognitive deficits induced by hypertension (Tedesco et al., 1999). Similarly, losartan was found to improve memory in a comparative study between losartan and atenolol (Fogari et al., 2003). The Study on COgnition and Prognosis in the Elderly (SCOPE) is a double-blind and placebo-controlled study of candesartan conducted in 4937 mild-to-moderate hypertensive patients, aged between 70 and 89 years with a mean follow-up of 3.7 years. It was found that in patients with low cognitive function, decline of the mini-mental state exam score over time was less in the candesartan group (Skoog et al., 2005). Furthermore, candesartan was shown to be associated with less decline in attention and episodic memory during a follow up period of 44 months in a substudy of the SCOPE (Saxby et al., 2008). Consistent with the results of SCOPE trial, another study showed that trajectories of cognitive decline evaluated with periodic mini-mental state exam were less steep with antihypertensive administration, and interestingly, patients taking ARBs even had improved cognitive scores (Hajjar et al., 2005). On the other hand, ARBs were found to reduce the incidence and progression of AD and dementia when compared to ACEIs and other cardiovascular drugs in 819,491 predominantly male participants (98%) aged 65 or older with cardiovascular disease (Li et al., 2010). A double-blind randomized controlled clinical trial called antihypertensives and vascular, endothelial, and cognitive function (AVEC) trial compared 1-year treatment of lisinopril, candesartan, or hydrochlorothiazide with regard to their effects on memory, executive function, CBF, and central endothelial function. This study found that ARBs are associated with improvement in executive function in hypertensive older adults with early executive cognitive impairment (Hajjar et al., 2009, 2012). A secondary longitudinal data analysis of the Ginkgo Evaluation of Memory Study showed that diuretic, ARB, and ACEI uses were, in addition to and/or independently of mean systolic blood pressure, associated with reduced risk of AD in older adults with normal cognition (*n* = 1,928) while only diuretic use was associated with reduced risk in participants with MCI (*n* = 320) (Yasar et al., 2013). Furthermore, a meta-analysis of studies on AD and aging showed that ARBs have a protective role in the risk of cognitive impairment of aging and AD, while both ACEIs and ARBs have benefits on prevention of AD (Zhuang et al., 2016). A more recent retrospective cohort study found that ARBs combined with statins reduced AD more than statins combined with non-RAS-acting antihypertensives (Barthold et al., 2020). Consistent with previous studies, ARBs were found to be more effective than ACEIs in reducing dementia risk. Similarly, a meta-analysis of 2 case-control studies and 7 cohort studies showed that ARB use was associated with a reduced risk of incident AD (Oscanoa et al., 2020). There are also ongoing studies which aim to further elucidate the role of ARBs on AD (Kehoe et al., 2018; Hajjar, 2020).

#### Studies Showing Neutral or Negative Effects on Cognition and Neural Protection

In contrast to many promising results of ARBs, there are also conflicting animal and human studies that did not demonstrate any beneficial effects of RAS-acting drugs. For example, some animal studies showed that losartan alone did not cause any changes in learning and memory (Kułakowska et al., 1996) whereas valsartan abolished the intracerebroventricular Ang II-induced improvement of memory retrieval and consolidation in animals (Braszko, 2005). Similar dissociation results were obtained in human studies as well. The SCOPE trial mentioned in the previous section did not show any difference between candesartan and placebo groups with regard to incidence of dementia (Lithell et al., 2003). The Ongoing Telmisartan Alone and in Combination with Ramipril Global Endpoint Trial (ONTARGET), a double-blind randomized controlled trial conducted on 25,620 participants, and the parallel Telmisartan Randomized Assessment Study in ACE Intolerant Subjects with Cardiovascular Disease (TRANSCEND) trial in 5,926 participants did not show any significant effect of ARBs on cognitive outcomes (Anderson et al., 2011). A quantitative meta-analysis of longitudinal studies was conducted to compare subjects with (*n* = 32,658) and without (*n* = 36,905) antihypertensive medication use (Chang-Quan et al., 2011). The study found that the risk of AD is unchanged even though risk of vascular dementia decreased with antihypertensive medication use.

### Angiotensin Converting Enzyme Inhibitors

#### Studies Showing Positive Effects on Cognition and Neural Protection

The ACEIs can improve basal learning performance, and antagonize scopolamine-induced learning deficits in animals (Barnes et al., 1992). Different cognitive effects of ACEIs in animal models were studied over the years. In the setting of diabetic rats with learning impairment, enalapril treatment improved water maze performance and hippocampal LTP (Manschot et al., 2003). Cilazapril improved memory in aged rats when administered at low doses without antihypertensive effects (Hirawa et al., 1999). Bhat et al. (2016) showed the anti-inflammatory effects of perindopril in rodent glial cells. Captopril slowed the accumulation of Aβ plaques and hippocampal ROS in a mouse model of AD (AbdAlla et al., 2013). Intranasal captopril treatment regulated microglial activation and decreased Aβ burden in an AD mouse model (Asraf et al., 2018). Although ACEIs have effects on varied brain functions, their activities within the brain were found to be different: enalapril and ramipril produced no significant inhibition of ACE at any time while captopril and zofenopril had modest, short-lasting effects, and lisinopril had long-lasting inhibitory effects (Cushman et al., 1989). In this regard, ACEIs are classified as central-acting and non-central-acting. Central-acting ACEIs are perindopril, captopril, fosinopril, lisinopril, trandolapril, and zofenopril, while benazepril, enalapril, moexepril, quinapril, and ramipril are non-central-acting (Sink et al., 2009).

There are also many human studies showing positive effects of ACEI use. A review by Amenta et al. (2002) suggested that calcium channel blockers and ACEIs had positive effects on cognitive domains of hypertension compared to diuretics and beta-blockers. The Perindopril Protection Against Recurrent Stroke Study (PROGRESS), a randomized, double-blind, placebo-controlled trial, showed reduced risk of dementia and cognitive decline with perindopril and indapamide in patients with history of prior stroke or transient ischemic attack (Tzourio et al., 2003). Furthermore, among patients with vascular disease or diabetes plus an additional risk factor in the Heart Outcomes Prevention Evaluation (HOPE) study, ramipril reduced the incidence of stroke and was associated with lower prevalence of cognitive impairment (Bosch et al., 2002). In a study evaluating effects of different antihypertensives drug classes in elderly patients with a blood pressure of less than 150/90 mmHg, it was shown that central-acting ACEIs were associated with a significantly lower incidence of AD compared to non-central-acting ACEIs, calcium channel blockers, beta-blockers, and diuretics (Ohrui et al., 2004a). Ohrui et al. (2004b) designed a randomized study to investigate the use of ACEIs as a possible treatment for mild-to-moderate AD, in addition to cholinesterase inhibitor. In this study, mini-mental state examination score decline was significantly reduced in AD patients treated with central-acting ACEI compared to other drug classes (Ohrui et al., 2004b). Consistent with these results, the prescription of ACEIs was found to be independently associated with the stability of cognitive function after 1-year follow-up of mild cognitive impairment patients (Rozzini et al., 2006).

These findings were further expanded by a substudy of the Cardiovascular Health Study showing that central-acting ACEIs are associated with lower risk of cognitive decline while non-central acting ACEIs were associated with a greater risk of incident dementia (Sink et al., 2009). As an explanation, it was hypothesized that central-acting ACEIs act via mechanisms other than blood pressure control (Sink et al., 2009). Additionally, centrally active ACEIs were associated with a reduced rate of cognitive decline in dementia patients and improved cognitive scores in the first 6 months after treatment (Gao et al., 2013). Interestingly, a recent pharmacogenetic study suggested that ACEIs can slow cognitive decline independently of blood pressure variations in patients with AD, particularly for APOE4-carriers of specific ACE genotypes (de Oliveira et al., 2018).

#### Studies Showing Neutral or Negative Effects on Cognition and Neural Protection

As with ARBs, there are studies reporting conflicting results with respect to effects of ACEIs on cognition. In one of the earliest studies, no cognitive and CBF changes were seen in patients treated with ceranapril for 4 weeks (Weiner et al., 1992). The Hypertension Old People in Edinburgh (HOPE) study evaluated cognitive function after treatment with ACEI (captopril) or diuretic (bendrofluazide) and found no difference in cognitive function after 24 weeks (Starr et al., 1996). Similar results were seen after 18 weeks of perindopril treatment (Louis et al., 1999). Although the double-blind placebo-controlled Systolic Hypertension in Europe (Syst-Eur) trial showed that antihypertensive treatment was associated with a lower incidence of dementia in elderly people (Forette et al., 1998), its follow-up study showed that calcium channel blocker therapy was effective protection against dementia in older patients regardless of enalapril use (Forette et al., 2002). Consistent with previous studies, the Cache County Study showed that ACEIs have no specific effects on AD risk apart from the general positive effects of antihypertensive medications on incidence of AD (Khachaturian et al., 2006). In the Rotterdam study, subjects (*n* = 2015) taking antihypertensive medication at baseline had a reduced incidence of dementia that was significant for vascular dementia and not significant for AD (in’t Veld et al., 2001). However, follow-up study of it showed that antihypertensive use reduced risks of all dementia including AD, with duration of hypertensive use being an important determinant (Haag et al., 2009). The same study did not find any apparent differences among different types of antihypertensive drugs (Haag et al., 2009). Finally, an observational study showed that ACEIs increase the risk of mortality in AD patients (Kehoe et al., 2013). However, this finding was not replicated in other cohorts (Lebouvier et al., 2020).

### Renin Inhibitors

Another potential modulator of the RAS-system is the class of drugs that directly inhibit renin. Aliskiren, a renin inhibitor, blocks the catalytic effects of renin, which is the first and rate-limiting step of the RAS. Besides its anti-hypertensive effects, it also promotes neuroprotection, improving functional outcomes in a model of ischemic stroke (Panahpour et al., 2019). Aliskiren pretreatment attenuated oxidative stress, glial activation, white matter lesion, and spatial working memory deficits by inhibiting brain renin (Dong et al., 2011). Additionally, aliskiren was shown to counteract pathophysiological mechanisms of AD in animal models. In particular, suppressed Aβ neurotoxicity and attenuated Aβ-induced intra-neuronal renin expression in rat cortical neurons (Chen et al., 2012). Furthermore, aliskiren also improved scopolamine-induced amnesia and increased acetylcholine through a decrease in acetylcholinesterase activity (Anil Kumar et al., 2015). However, a double-blind placebo-controlled trial showed that the incidence rate of dementia did not change in people over 80 years old who were treated with aliskiren for high blood pressure (Peters et al., 2008). Aliskiren is a promising agent for prevention or treatment of dementia (O’Caoimh et al., 2014). However, more clinical trials with longer follow-up periods are needed to investigate effects of DRIs on cognition comprehensively.

## Conclusion

Alzheimer’s disease and frailty are interrelating age-related disorders with a high level of morbidity and mortality. Although epidemiological and observational studies have shown close associations of frailty with AD, the biological mechanisms responsible for linking these two conditions remain elusive. Mitochondrial dysfunction, chronic inflammation, and oxidative stress constitute primary theories of aging and have been implicated as major contributors to the pathogenesis of both frailty and AD. The renin–angiotensin system is a central hormonal system that contributes to both inflammation and mitochondrial dysfunction. Many important studies have emerged that suggest that angiotensin system blocking drugs, commonly used in clinical practice for hypertension and heart failure, can favorably impact many chronic disease states, tissues and organ systems that are negatively impacted by age and inflammation. The choice of RAS-blocking drug and deciding on the onset of treatment remain uncharted territory.

## Author Contributions

All authors listed have made a substantial, direct and intellectual contribution to the work, and approved it for publication.

## Conflict of Interest

The authors declare that the research was conducted in the absence of any commercial or financial relationships that could be construed as a potential conflict of interest.
